# The Association Between Peri-Hemorrhagic Metabolites and Cerebral Hemodynamics in Comatose Patients With Spontaneous Intracerebral Hemorrhage: An International Multicenter Pilot Study Analysis

**DOI:** 10.3389/fneur.2020.568536

**Published:** 2020-10-26

**Authors:** Frank Rasulo, Simone Piva, Soojin Park, Mauro Oddo, Murad Megjhani, Danilo Cardim, Ilaria Matteotti, Leonardo Gandolfi, Chiara Robba, Fabio Silvio Taccone, Nicola Latronico

**Affiliations:** ^1^Department of Anesthesia, Intensive Care and Emergency Medicine, Spedali Civili University Hospital of Brescia, Brescia, Italy; ^2^Department of Neurocritical Care, Columbia University, Presbyterian Hospital Medical Center of New York, New York City, NY, United States; ^3^Department of Intensive Care, Canton of Vaud University Hospital (CHUV)-Lausanne University Hospital, University of Lausanne, Lausanne, Switzerland; ^4^Department of Neurology and Neurotherapeutics, University of Texas Southwestern Medical Center, Dallas, TX, United States; ^5^Department of Anesthesia and Intensive Care, Ospedale Policlinico San Martino Istituto di Ricovero e Cura a Carattere Scientifico (IRCCS), Genova, Italy; ^6^Department of Intensive Care, Erasme Hospital, Brussels, Belgium

**Keywords:** autoregulation cerebral compliance, intracerebal hemorrhage, metabolism, hemodynamics, microdialyis

## Abstract

**Background and Objective:** Cerebral microdialysis (CMD) enables monitoring brain tissue metabolism and risk factors for secondary brain injury such as an imbalance of consumption, altered utilization, and delivery of oxygen and glucose, frequently present following spontaneous intracerebral hemorrhage (SICH). The aim of this study was to evaluate the relationship between lactate/pyruvate ratio (LPR) with hemodynamic variables [mean arterial blood pressure (MABP), intracranial pressure (ICP), cerebral perfusion pressure (CPP), and cerebrovascular pressure reactivity (PRx)] and metabolic variables (glutamate, glucose, and glycerol), within the cerebral peri-hemorrhagic region, with the hypothesis that there may be an association between these variables, leading to a worsening of outcome in comatose SICH patients.

**Methods:** This is an international multicenter cohort study regarding a retrospective dataset analysis of non-consecutive comatose patients with supratentorial SICH undergoing invasive multimodality neuromonitoring admitted to neurocritical care units pertaining to three different centers. Patients with SICH were included if they had an indication for invasive ICP and CMD monitoring, were >18 years of age, and had a Glasgow Coma Scale (GCS) score of ≤8.

**Results:** Twenty-two patients were included in the analysis. A total monitoring time of 1,558 h was analyzed, with a mean (SD) monitoring time of 70.72 h (66.25) per patient. Moreover, 21 out of the 22 patients (95%) had disturbed cerebrovascular autoregulation during the observation period. When considering a dichotomized LPR for a threshold level of 25 or 40, there was a statistically significant difference in all the measured variables (PRx, glucose, glutamate), but not glycerol. When dichotomized PRx was considered as the dependent variable, only LPR was related to autoregulation. A lower PRx was associated with a higher survival [27.9% (23.1%) vs. 56.0% (31.3%), *p* = 0.03].

**Conclusions:** According to our results, disturbed autoregulation in comatose SICH patients is common. It is correlated to deranged metabolites within the peri-hemorrhagic region of the clot and is also associated with poor outcome.

## Introduction

Spontaneous intracerebral hemorrhage (SICH) accounts for almost 15% of all strokes worldwide, with the incidence rates of primary SICH in low- and middle-income countries being twice the rates compared to high-income countries (22 vs. 10 per 100,000 persons/year) in 2000–2008, and with case fatality rates of 30–48% in low- to middle-income countries and 25–30% in high-income countries (1, 2). The outcome of these patients is multifactorial, and poor outcomes are related to both metabolic and hemodynamic derangements. In fact, following SICH-induced acute brain injury, an imbalance of consumption, utilization, and delivery of oxygen and glucose, along with intracranial hypertension and a reduced cerebral perfusion pressure (CPP) which accompanies hematoma expansion, are responsible for secondary injuries (3–6). Monitoring these parameters in order to properly direct treatment is possible with cerebral microdialysis (CMD).

CMD, first introduced in 1966, aids clinicians in better understanding brain tissue metabolism in intensive care settings (7, 8). In particular, variations in the lactate/pyruvate ratio (LPR) may correspond to a mismatch between oxygen delivery and utilization, also influenced by hemodynamic variables.

Control of hemodynamic variables after SICH is in fact paramount in avoiding secondary brain injury. There is consensus regarding the fact that the initial dimension and growth of the hematoma can cause both primary and secondary tissue damage. The latter may be the consequence of a series of complications such as ischemia, brain edema, development of apoptotic processes, and toxic effects due to the degraded components of hemoglobin and complement activation (8). Additionally, although the exact pathophysiological mechanisms remain undetermined, cerebrovascular autoregulation (CA) derangement could play an important role in promoting additional brain injury, especially in the peri-hemorrhagic penumbra region, thus affecting outcomes (9–11).

Based on the above-mentioned hypothesis, this study sought to evaluate the association between LPR and the hemodynamic [mean arterial blood pressure (MABP), intracranial pressure (ICP), CPP, and CA expressed by cerebrovascular pressure reactivity (PRx)] and metabolic variables (glutamate, glucose, and glycerol) within the peri-hemorrhagic penumbra region of the blood clot in comatose patients with SICH. Moreover, it explored the association of metabolic and hemodynamic variables with outcome.

## Methods

This was a multicenter cohort study regarding a retrospective dataset analysis of non-consecutive comatose patients who underwent multimodal monitoring following SICH. The study took place between April 2014 and June 2017 within three neurocritical care units: Spedali Civili University Hospital of Brescia, Columbia University New York Presbyterian Hospital Medical Center of New York, and the CHUV-Lausanne University Hospital and University of Lausanne, Switzerland. Patients were included if they: (1) had a diagnosis of SICH; (2) had an indication for invasive ICP and CMD monitoring; (2) were comatose with a Glasgow Coma Scale (GCS) score of ≤8 at enrollment; and (3) were >18 years old.

Patients were excluded if there were any contraindications for invasive intracranial catheter placement (e.g., an INR >2 or a platelet count <100,000/mL), moribund patients, and age <18 years.

This study was conducted in accordance with the Declaration of Helsinki and approved by the Brescia Ethics Committee. In Brescia, patients' informed consent was waived due to the fact that the Italian legislation lacks a clear definition of what is considered a legal representative of temporarily incapacitated adult patients (12). Informed consent was therefore obtained from the patients once, and if, they regained mental competency. Regarding the Lausanne center, waiver of consent was authorized since the study consists of a retrospective dataset analysis of standard-of-care procedures, and at the Columbia center, the University Institutional Review Board approved the collection and analysis of data and a data-sharing agreement was executed.

The STROBE (Strengthening the Reporting of Observational Studies in Epidemiology) guidelines were followed for reporting the results of this cohort study (13).

### Patient Management

All patients were intubated and mechanically ventilated during the entire monitoring period, and arterial blood gas samples were obtained every 2 h in order to maintain a target range of end-tidal carbon dioxide [etCO2] between 35 and 45 mmHg. Intravenous midazolam or propofol and fentanyl were used for analgo-sedation, and, if required, a muscle relaxant was added. Therapy was aimed at maintaining systolic arterial blood pressure (SABP) between 140 and 160 mmHg and a mean MABP <130 mmHg according to current guidelines (14). When necessary, antihypertensive or catecholamine therapy was used to reach the arterial blood pressure (ABP) target range. ICP and CPP were maintained respectively at <20 mm Hg and between 50 and 70 mmHg, depending also on PRx-based optimal CPP values. The diagnostic and therapeutic strategies for the patients enrolled were not modified by this study.

### Hemodynamic and Neuro-Monitoring

Systemic hemodynamic monitoring consisted of invasive ABP from the radial artery, continuous electrocardiography, and pulse oximetry (Edwards Life Sciences, Irvine, CA). For all three centers, the decision to apply the multimodality monitoring [invasive ICP and microdialysis (MD)] was made based on an individual case decision, according to the neurosurgeon, and on intensive discussion. Intracranial pressure monitoring was performed either by means of an intraparenchymal fiber-optic transducer (Camino Laboratories, Integra NeuroSciences, San Diego, CA) or through catheter insertion into the brain ventricles and connected to an external pressure transducer and drainage system (Codman, Johnson & Johnson Medical Ltd., Raynham, MA).

### Microdialysis

The MD catheters (membrane length, 10 mm; CMA 70,71 CMA Microdialysis, Sweden) were implanted either through tunneling or following burr hole and bolt insertion and were positioned within the brain tissue surrounding the clot, corresponding to the metabolic/ischemic penumbra. To ensure correct placement of the probes within the peri-hematomal region, a brain CT scan was performed following CMD catheter placement. The catheters were perfused with artificial cerebrospinal fluid at a rate of 0.3 μL/min and the samples were collected in microvials for analysis at 1- to 2-h intervals immediately after collection. The samples were analyzed for glucose, pyruvate, lactate, glutamate, and glycerol using the ISCUS Clinical Microdialysis Analyzer (CMA Microdialysis, Sweden). The first sample obtained following catheter placement was discarded since the measurements may have been altered by tissue trauma induced by the catheter insertion itself.

### Multimodal Monitoring

For multimodal data acquisition and calculation of the derived indices, we used the Intensive Care Monitoring software system (ICM+^®^, Cambridge Enterprise, UK) running on bedside laptop computers. Simultaneous recordings of analog signals from the ICP and ABP signals were digitalized with an analog-to-digital converter, sampled (sampling at 50 Hz) and averaged every 5 s. Artifacts were manually detected and removed. In the Brescia and New York centers, CA assessment was measured continuously through the calculation of the PRx every 60 s as a Pearson's correlation coefficient between 30 consecutive samples of the mean arterial pressure and ICP and was evaluated as a variable index, changing in time along with ICP, CPP, and arterial pressure, as described by Smielewski et al. (15). This coefficient represents an index of covariance ranging from −1 to 1; a PRx >0.2 is indicative of a defective CA (16). Regarding the Lausanne center, PRx was not available; therefore, the correlations between the mean arterial pressure and ICP were calculated *a posteriori* after the data were derived retrospectively from the patients' database. We adopted this same threshold to define defective CA because no specific threshold was available for SICH patients during the time this study was conducted. The data acquired from the patients' MD values in relation to CA were used for research purposes only and did not influence the clinician's therapeutic strategy.

### Outcome

The primary outcome was to evaluate the correlation between LPR and metabolic and hemodynamic variables, measured in the peri-hemorrhagic area of the SICH. The secondary outcomes were the Glasgow Outcome Scale (GOS) score and mortality at 6 months, obtained retrospectively.

### Statistical Analysis

The data derived from the CMD analyzer were visualized through the LAB Pilot software (Solna, Sweden). For each patient, the consecutive values of pyruvate, lactate, glycerol, glutamate, and glucose were gathered and the LPR was derived. The data obtained were transferred to an Excel spreadsheet containing the registration of MAP, ICP, CPP, and PRx every minute. The hourly mean hemodynamic variables (MAP, ICP, CPP, and PRx) were compared to the metabolic variables acquired every hour with CMD.

We expressed continuous variables as mean (standard deviation, SD) for the normally distributed variables or median (interquartile range, IQR) for the non-normally distributed variables. Qualitative variables were expressed as frequencies and percentages.

To compare the different collected covariates between centers, a linear mixed-effects model (package lme4 in R) has been used to compare continuous variables in order to take into account the repeated patient measurements nested in each center, whereas ordered logistic regression for repeated measures was used to compare the GOS scores.

Concerning the unadjusted analysis, LPR was firstly dichotomized into normal and abnormal values (cutoffs of 25 and 40), as well as PRx (normal value ≤0.2), and a generalized mixed-effects model with a single variable was used including both CMD and the hemodynamic covariates. Alternatively, the dichotomized LPR and PRx were considered as dependent variables and each CMD and hemodynamic covariate as independent variables, and patients were considered nested in the center as a random effect (package nlme).

Lme4 R package was used to perform an adjusted analysis using a linear mixed-effects model of the relationship between LPR and MD and CA variables, adjusted for age and admission GCS (17). An empty model was first performed (using only the intercept for LPR), and then the fixed effect was then added followed by the random effects. As a fixed effect, PRx was entered first, followed by glucose, glutamate, glycerol, age, and GCS. As a random effect, the intercept for patients nested in the centers was added. ANOVA comparison between all the resulted models was performed in order to choose the final model, for which the *p-*values for fixed effect were obtained through Satterthwaite's method (lmerTest package).

For patients' outcomes (mortality and GOS), we first dichotomized GOS into unfavorable (GOS ≤ 3) and favorable (GOS > 3). In order to see whether there were any differences in the hemodynamic or CMD covariates, we ran an unadjusted analysis using a linear mixed model. The dichotomized GOS or mortality was used as a fixed effect, each of the hemodynamic or CMD covariates as dependent variables, and patients nested in the center as random effects. Verification of the results was performed with ANOVA for repeated measures with the function aov in R.

## Results

The multimodal monitoring data were obtained from a total monitoring time of 1,558 h, with a mean (SD) monitoring time of 70.72 h (66.25 h) per patient. There were a total of 1,901 readings and an average of 79 readings per patient.

Patients' characteristics are represented in Table 1, divided by the participating centers. There was no statistically significant difference in the demographics, hemodynamic variables (PRx, MABP, and ICP), and the MD data between centers. Except for the admission GCS, there was a difference concerning the outcome variables between the two centers which provided GOS data (it was not possible to obtain GOS data from one of the three centers).

**Table 1:** Demographic, hemodynamic, microdialysis, and outcome data divided by centers.

**Variables**	**Columbia**	**Italy**	**Lausanne**	**Total mean values**	***p*-Value**
	**(*n* = 8)**	**(*n* = 9)**	**(*n* = 5)**		
Age, mean, SD (years)	74.75 (6.80)	73.11 (5.90)	53 (17.23)	67.88 (13.61)	0.0064
Admission GCS, median (IQR)	7 (5)	3 (1)	4 (2)	4 (4)	0.001
**HEMODYNAMIC DATA**					
PRx, mean (SD)	0.04 (0.23)	0.15 (0.24)	−0.016 (0.307)	0.036 (0.284)	0.130
MABP, mean (SD)	112.08 (18.22)	99.77 (15.28)	87.24 (7.89)	96.73 (16.80)	0.926
ICP, mean (SD)	14.01 (9.58)	12.61 (7.23)	10.88 (5.59)	12.06 (7.27)	0.195
**MICRODIALYSIS DATA**					
LPR	35.27 (18.14)	25.05 (14.15)	36.43 (17.22)	33.263	0.833
Lactate	4.83 (1.77)	3.393 (2.23)	6.14 (3.97)	5.021	0.855
Pyruvate	144.71 (56.00)	132.96 (23.19)	167.92 (62.30)	151.23	0.837
Glucose	0.82 (0.57)	2.05 (1.19)	1.38 (0.70)	1.346	0.833
Glutamate	40 (2.65)	34.62 (19.41)	63.61 (71.63)	50.06	0.728
Glycerol	63 (4.35)	110.20 (139.25)	244.97 (180.79)	193.27	0.051
**OUTCOMES**					
GOS at 6 months, median (IQR)	NA	3 (2)	3 (2)	3 (2)	0.212
Survivors, *n* (%)	2 (25%)	3 (33%)	2 (40%)	7 (32%)	0.918

*GCS, Glasgow Coma Scale; PRx, cerebrovascular pressure reactivity; MABP, mean arterial blood pressure; ICP, intracranial pressure; LPR, lactate/pyruvate ratio; GOS, Glasgow Outcome Scale; NA, not available*.

**Figure 1:**
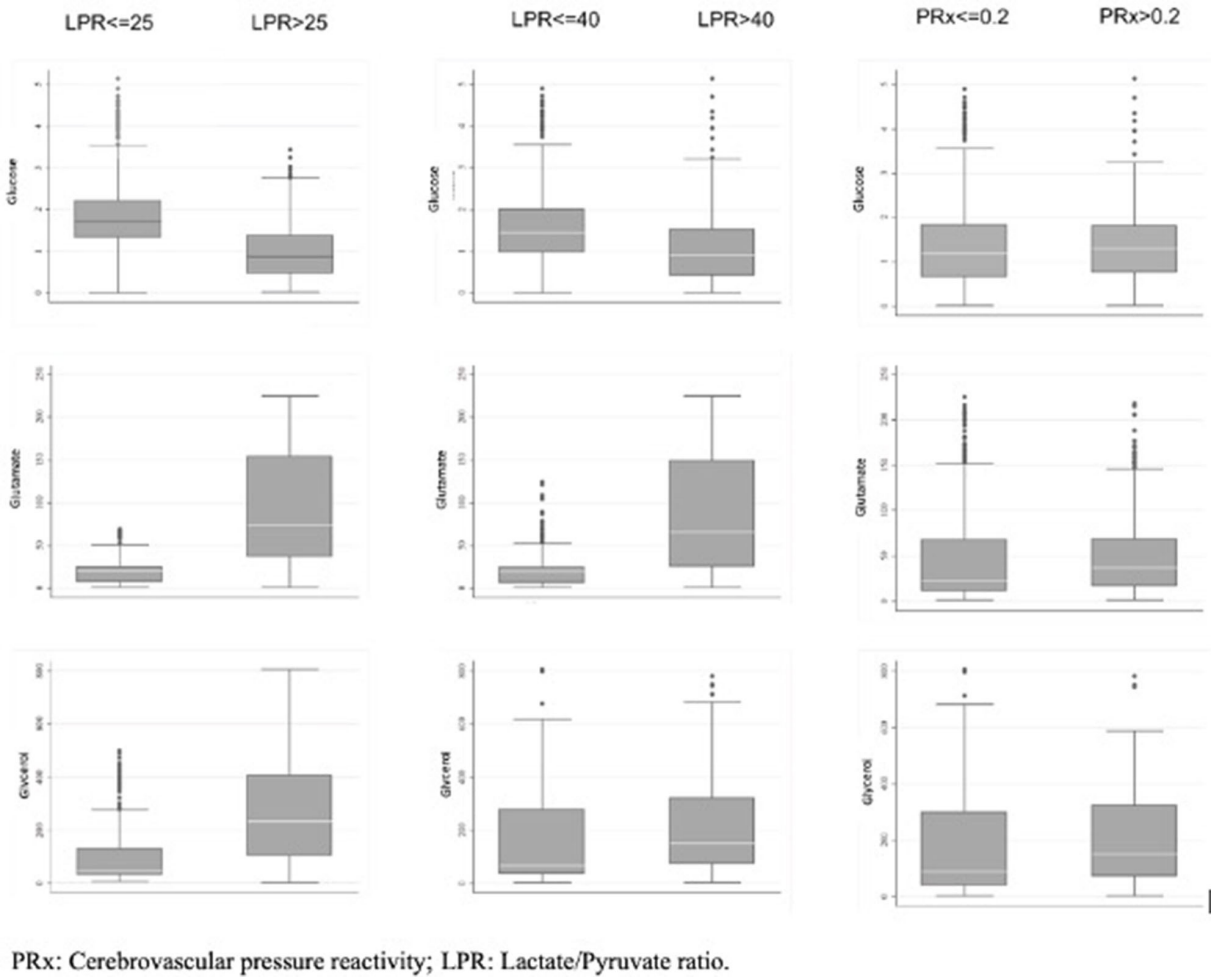
Box plot of the MD variables collected and their correlation to abnormal and normal level of LPR and PRx.

**Figure 2:**
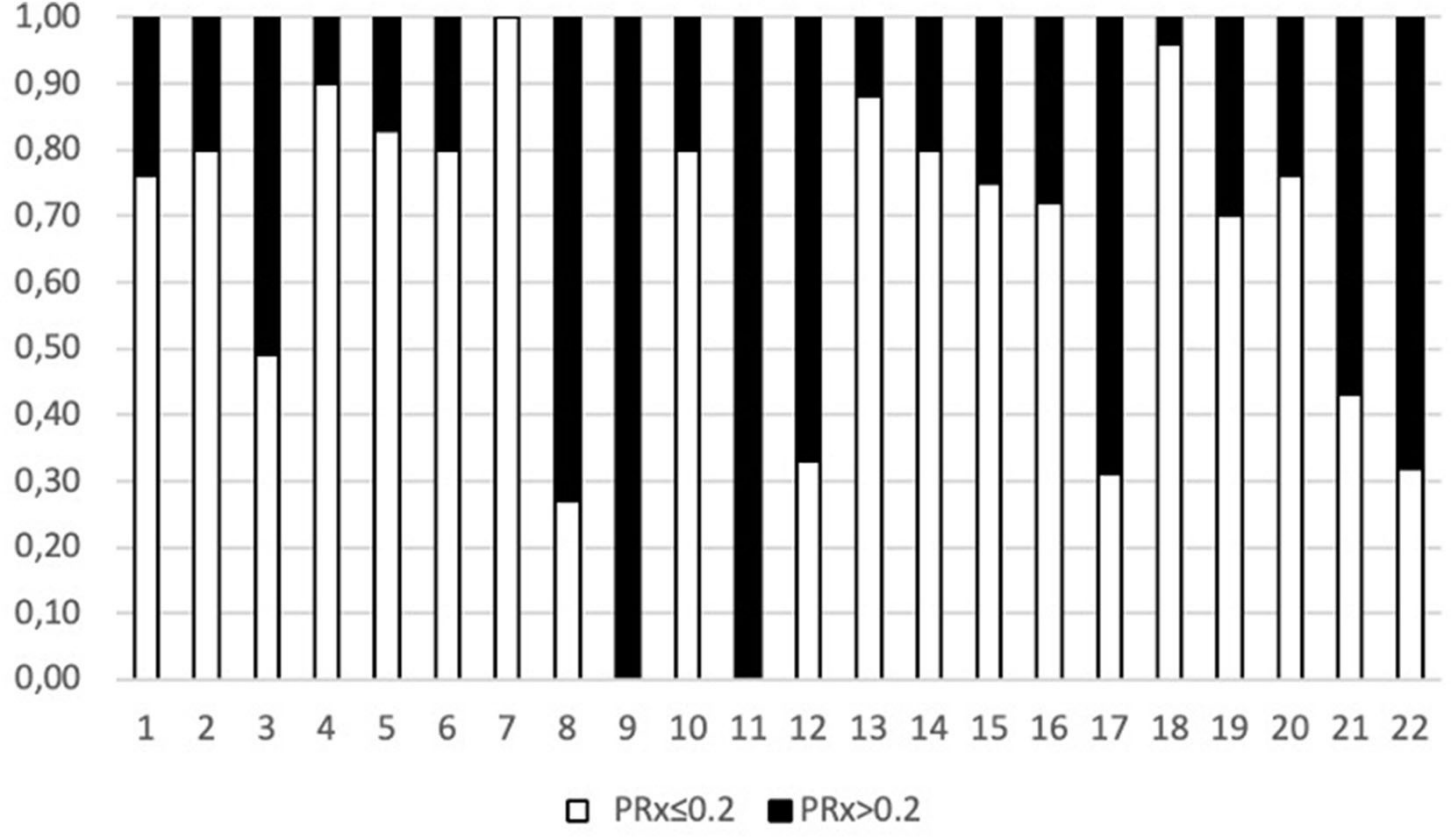
Representation of percentage of time in which each patient had an abnormal PRx (>0.2).

When considering a dichotomized LPR (25 or 40), except for glycerol, there was a statistically significant difference in all the measured variables PRx, glucose, and glutamate (Table 2 and Figure 1). When dichotomizing PRx as the dependent variable, the unadjusted logistic mixed model shows LPR as the only CMD variable related to CA. Moreover, 21 out of the 22 patients (95%) had disturbed CA during the observation period (Figure 2). In the adjusted analysis using linear mixed-effects analysis, considering LPR as the dependent variable, the best model resulting from the ANOVA comparison was the one which included PRx, glucose, and glutamate (BIC = 3,585) as fixed effects and a random intercept for patients but not for centers (Table 3). Adding a slope random effect for patients and centers did not improve the model, and the ANOVA comparison between the null model with only random intercepts only and the final model yielded χ^2^ = 517.51, *p* < 0.0001. The correlation between PRx and LPR showed a significant variance in the intercepts across patients (SD = 8.66, 95% CI = 5.192–11.838), and visual inspection of the residual plots did not reveal any obvious deviation from homoscedasticity or normality. Worth noting is that, although the LPR difference between PRx <0.2 and >0.2 is statistically significant [33.33 (16.56) vs. 33.08 (19.74)], it is minimal and, therefore, may suggest that the PRx threshold chosen may not be the most clinically relevant in this population.

**Table 2:** Unadjusted analyses for pathological and non-pathological LPR ratios with two different cutoffs and pathological and non-pathological PRx.

	**≤25 (*n* = 383)**	**>25 (595)**	***p*-Value**	**≤40 (518)**	**≥40 (460)**	***p*-Value**
**Pathological and non-pathological LPR ratios**						
PRx	0.008 (0.3)	0.076 (0.25)	0.0387	0.026	0.086	<0.0001
Glucose, mean (SD) (mmol/L)	1.85 (1.04)	1.00 (0.65)	<0.0001	1.57 (0.93)	0.68 (0.50)	<0.0001
Glutamate, mean (SD) (μmol/L)	21.22 (17.78)	90.80 (64.96)	0.001	25.15 (23.77)	126.56 (56.18)	<0.0001
Glycerol, mean (SD) (μmol/L)	115.00 (131.77)	267.75 (185.09)	0.218	175.08 (168.39)	253.17 (196.94)	0.6750
	**≤0.2**			**>0.2**		***p*****-Value**
**Pathological and non-pathological PRx**						
LPR	33.33 (16.56)			33.08 (19.74)		0.039
Glucose, mean (SD) (mmol/L)	1.35 (0.96)			1.34 (0.86)		0.903
Glutamate, mean (SD) (μmol/L)	49.37 (58.16)			51.59 (50.14)		0.085
Glycerol, mean (SD) (μmol/L)	185.61 (182.75)			209.56 (167.99)		0.816

*PRx, cerebrovascular pressure reactivity; LPR, lactate/pyruvate ratio*.

**Table 3:** Adjusted linear mixed-effects model for lactate/pyruvate ratio (LPR).

**Variables**	**β**	**SE β**	**95% CI (β)**	***p-*Value**
Prx	1.65	1.05	−0.40 to 3.72	0.022
Glucose	−4.69	0.70	−5.70 to −3.69	<0.0001
Glutamate	0.15	0.015	0.12–0.19	<0.0001

*The fixed effect coefficient for the final mixed model are represented along with the p-value*.

Regarding outcomes, surviving patients had a statistically significant lower PRx and spent on average less time with an abnormal PRx compared to patients who did not survive (27.9% of the total time vs. 56.0%, *p* = 0.03). Surviving patients had lower glycerol values, whereas the glucose level and the mean ABP (both higher in surviving patients) were at the limit of significance (Table 4).

**Table 4:** Unadjusted analysis of measured outcomes.

	**Outcomes**				***p-*****Value**	
	**Death, *n* = 7 (mRS = 6, GOS = 1)**	**Alive (*n* = 15)**	**Unfavorable GOS (*n* = 12)**	**Favorable GOS (*n* = 2)**	**Dead vs. alive**	**Favorable vs. unfavorable**
PRx, mean (SD)	0.12 (0.22)	0.03 (0.12)	0.085 (0.28)	−0.12 (0.17)	0.003	0.167
MABP, mean (SD) (mmHg)	82.77 (30.58)	97.80 (11.32)	95.31 (9.33)	88.80 (5.77)	0.071	0.878
ICP, mean (SD) (mmHg)	20.82 (26.17)	11.26 (4.92)	6.34 (4.74)	11.75 (4.590)	0.182	0.754
L/P ratio	30.44 (19.24)	34.52 (16.56)	32.73 (17.15)	18.28 (1.00)	0.092	0.437
Glucose, mean (SD) (mmol/L)	1.35 (0.57)	1.38 (1.10)	1.55 (0.82)	4.149 (0.23)	0.057	0.011
Glutamate, mean (SD) (μmol/L)	30.18 (27.09)	63.01 (64.72)	17.98 (1.49)	52.53 (57.20)	0.718	0.616
Glycerol, mean (SD) (μmol/L)	271.02 (191.96)	149 (97.9)	29.90 (1.23)	201.36 (178.65)	0.009	0.412

*PRx, cerebrovascular pressure reactivity; MABP, mean arterial blood pressure; ICP, intracranial pressure; LPR, lactate/pyruvate ratio; GOS, Glasgow Outcome Scale (unfavorable, GOS ≤ 3)*.

## Discussion

In this multicenter retrospective observational cohort study performed in patients suffering from SICH, we confirmed the hypothesis that alterations in LPR may be correlated to the disturbance of CA (expressed as a PRx > 0.2) and to the glucose and glutamate levels. Patients who survived had a statistically significant lower PRx, spent less time with an altered PRx, and had lower glycerol and higher glucose values. Those who died had a higher LPR, higher ICP, and lower MABP values, although not statistically significant (Table 4 and Figure 1).

For spontaneous intracerebral hemorrhage (ICH), there are only limited data available on dynamic autoregulation and CPP management, and so far, there are no data regarding autoregulation-based CPP management (18, 19). There was a statistically significant correlation between LPR and the measured variables (PRx, glucose, glutamate, and glycerol) present in both threshold levels of 25 and 40 (Table 2). Interestingly, the brain glucose concentrations were <1 mmol/L in the majority of patients with an LP ratio >25.

Multimodality monitoring in this study did not involve brain oxygenation (PbO_2_), which possibly would have provided further insight toward the understanding of the various interactions between the metabolic and hemodynamic variables. The results from one study where the ICH patients were monitored with both PbO_2_ and CMD suggest that this type of monitoring can demonstrate local derangements in the peri-hematoma cavity that are not reflected in global indices such as the PRx (20).

Recently, the existence of an ischemic penumbra in SICH patients has been challenged and a switch of concept from am ischemic to a metabolic penumbra was suggested, referring to the finding of an increased glucose metabolism in the peri-hemorrhagic region in the absence of ischemia (19, 21). The transient focal increases in glucose metabolism have been interpreted as signs of ongoing neuronal injury lasting for several days. Worthy of note is that the patients in our analysis with disturbed CA had the lowest glucose levels and those with a favorable outcome had higher values. The LPR was not independently correlated to the outcome.

The association between a low ABP and the outcome has been known for almost two decades (22, 23). Among the modifiable risk factors for SICH, arterial hypertension is the most frequent (24). Hypertension has been associated with early hematoma growth and poor outcomes in patients with spontaneous SICH (25). However, an excessively low ABP might cause cerebral hypoperfusion and ultimately lead to a poor outcome (24). Despite the important secondary outcomes found in recent trials, when attempting to establish the optimal ABP level following acute SICH, there was either a small benefit (INTERACT-2, Intensive Blood Pressure Reduction in Acute Cerebral Hemorrhage Trial) or no benefit (ATACH-2, Antihypertensive Treatment of Acute Cerebral Hemorrhage II Study) when intensive systolic ABP reduction was compared with modest or standard BP reduction (24, 26). The more recent meta-analyses including studies investigating this issue yielded similar conclusions: aggressive ABP control in the acute phase of SICH is not beneficial. The 2018 European Society of Cardiology/European Society of Hypertension Guidelines for the management of arterial hypertension do not recommend treatment to immediately lower ABP in patients with acute SICH and a careful lowering of systolic blood pressure (SBP) to <80 mmHg *via* intravenous infusion may be considered only in patients with SBP ≥ 220 mmHg (14).

Disturbed CA in patients with SICH may worsen the effect of an excessively low ABP by increasing the lower threshold of CA below which cerebral hypoperfusion and ischemia ensue (25). Since substrate supply depends on cerebral blood flow, patients suffering from an impaired global autoregulation may be more vulnerable to secondary brain injury.

This study shows that SICH patients with disturbed CA and low blood pressure may be at risk of hypoperfusion and, therefore, of poor outcomes. Previous literature suggests that optimal CPP thresholds may be higher in SICH patients, adding further support that target directed CPP thresholds should be based on patient-specific monitoring parameters in order to individualize therapy (27).

Although the association of low MABP and high LPR with poor outcome, and *vice versa*, was reproduced in our study, whether low MABP and disturbed CA occurred simultaneously is not known.

Many factors may reduce the cerebrovascular autoregulatory reserve; however, studies showing CA as a consequence of cerebral metabolism dysfunction are lacking (28). There is literature questioning the presence of an ischemic penumbra, hypothesizing of the existence of important metabolic derangements in the tissue surrounding the clot. Although our results do not confirm the existence of a metabolic penumbra, the presence of disturbed glucose utilization may be the result of metabolic derangements near this region (21, 29, 30).

### Limitations

The sample size was small, not predetermined, and the study included a highly selected population of SICH patients.

Some data comparing the different variables did not reach statistical significance. Therefore, these findings must be weighed critically, and only speculative conclusions can be drawn so far.

The study was an explorative retrospective analysis of non-consecutive patients pertaining to three different centers with data which were partially incomplete, for example the GOS, available in only two of the three centers. This study was not able to obtain sufficient data regarding the volume and size of the clot, which would have provided useful information regarding the neurological severity through grading scores such as the ICH score (31). Data regarding the APACHE or SAPS were also lacking from all three centers and therefore not included in the analysis.

The PRx threshold of 0.2, above which CA is considered to be altered, has been validated in traumatic brain injury (TBI) patients, but has not been extensively validated in patients with ICH (9).

Only the MABP was available for all three centers and not the SABP. However, MABP was fundamental in order to calculate PRx.

Finally, GOS was collected by chart review following telephone interview, therefore representing a retrospective collection of outcome.

Although accompanied by its limitations, this study helps set the base from which future large prospective trials may be designed, powered in order to confirm the results obtained. If so, multimodality monitoring which includes the measurement of metabolites and their correlation with CA would represent an important adjunct in avoiding secondary brain injury and help improve the outcomes in patients with SICH.

## Conclusions

With its limitations, this study demonstrates that disturbed CA in comatose SICH patients is frequent and is associated with deranged cerebral intraparenchymal metabolites. Moreover, a trend toward worse outcomes in patients with altered PRx was observed in our study.

## Data Availability Statement

The raw data supporting the conclusions of this article will be made available by the authors, without undue reservation.

## Ethics Statement

The studies involving human participants were reviewed and approved by Spedali Civili Comitato Etico. Written informed consent for participation was not required for this study in accordance with the national legislation and the institutional requirements.

## Author Contributions

FR, NL, SPi, and SPa contributed to the study conception and design. FR, NL, SPi, SPa, IM, LG, MO, MM, DC, CR, and FT performed the data analysis and interpretation. FR, SPa IM, LG, and MO enrolled the patients for each corresponding study center. FR, NL, SPi, SPa, MO, MM, DC, CR, and FT revised the manuscript for important intellectual content. FR and NL gave final approval of the version to be published. All authors read and approved the final manuscript. All authors contributed to the article and approved the submitted version.

## Conflict of Interest

The authors declare that the research was conducted in the absence of any commercial or financial relationships that could be construed as a potential conflict of interest.

## Abbreviations

CMD: cerebral microdialysis
SICH: spontaneous intracerebral hemorrhage
LPR: lactate/pyruvate ratio
MABP: mean arterial blood pressure
ICP: intracranial pressure
CPP: cerebral perfusion pressure
CA: cerebrovascular autoregulation
PRx: cerebrovascular pressure reactivity
GOS: Glasgow Outcome Scale.
